# Supplementary material for: Human MLH1/3 variants causing aneuploidy, pregnancy loss, and premature reproductive aging
Source: Nat Commun. 2021 Aug 18;12:5005. doi: 10.1038/s41467-021-25028-1 (PMC8373927; doi:10.1038/s41467-021-25028-1)
Supplement: Supplementary file 1 — Supplementary information [file 41467_2021_25028_MOESM1_ESM.pdf]

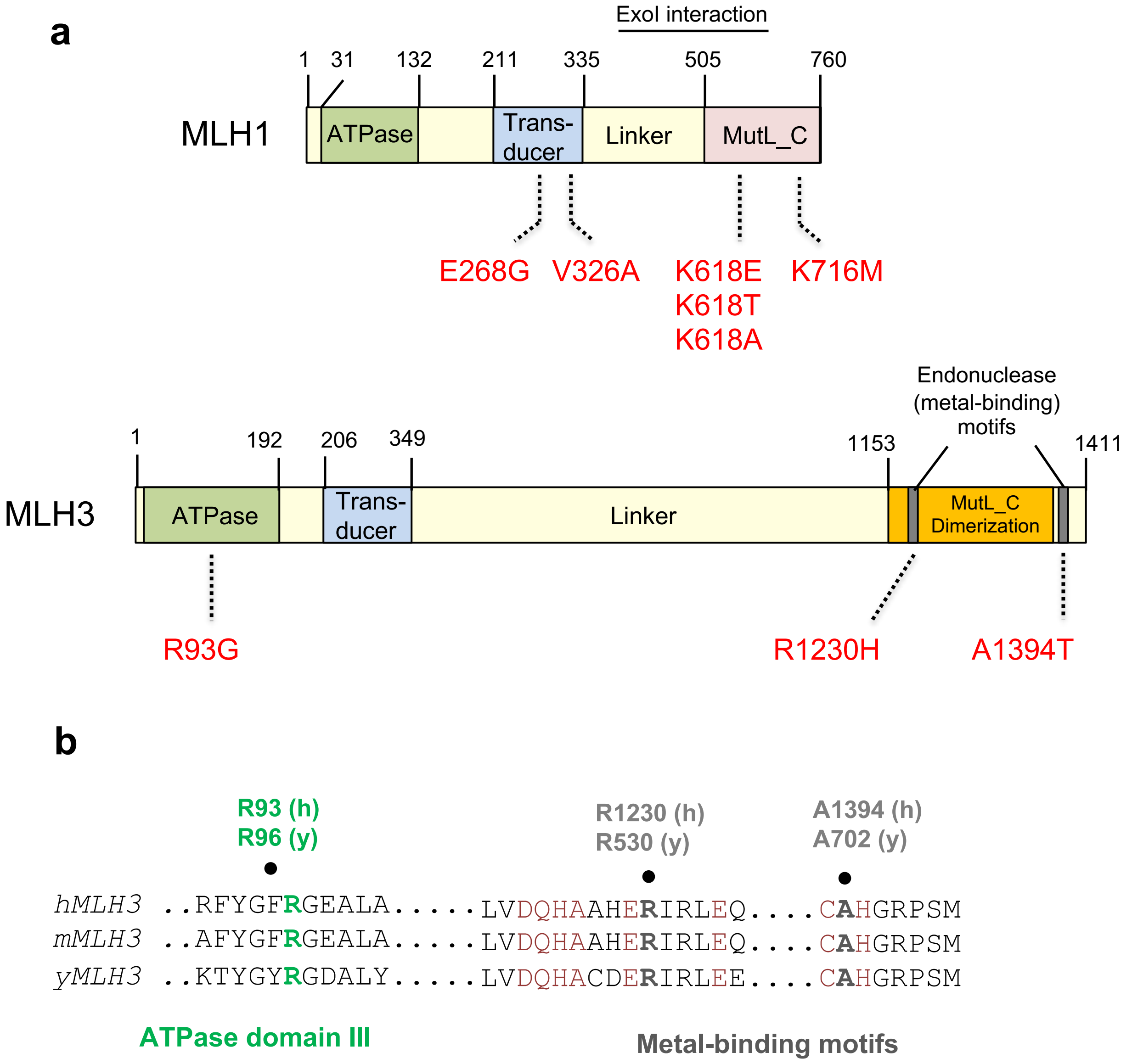

Supplementary Fig 1. **Cartoon of mouse MLH1 and MLH3 protein organization, and locations of variants modeled in mice.** **a** Amino acid (AA) positions in full-length isoforms, above the gene diagrams, correspond to the mouse proteins. The human variants modeled are noted in red below the protein diagrams, and those AA positions correspond the the human proteins. Indicated in the diagrams (colored boxes,except for the Exo[nuclease]I interaction domain) are approximate locations of domains in each protein. **b** Locations (black dots) of AA alterations modeled in conserved ATPase and endonuclease domains in yeast and mouse MLH3. h = human; m = mouse; y = yeast. The key Aas for the metal binding motifs are in red, based on homology to residues in yeast Pms1 that form a metal binding site in the C-terminal domain of yeast Mlh1-Pms1 (Gueneau et al. 2013). Conserved ATPase domains are based on (Ban and Yang 1998; Tran and Liskay 2000).

• Gueneau E, Dherin C, Legrand P, Tellier-Lebegue C, Gilquin B, Bonnesoeur P, et al. (2013) Nat Struct Mol Biol 20: 46-468.
• Tran PT, Liskay RM (2000). Mol Cell Biol 20: 6390–6398.
• Ban C. Yang W (1998). Cell 95: 541–552.

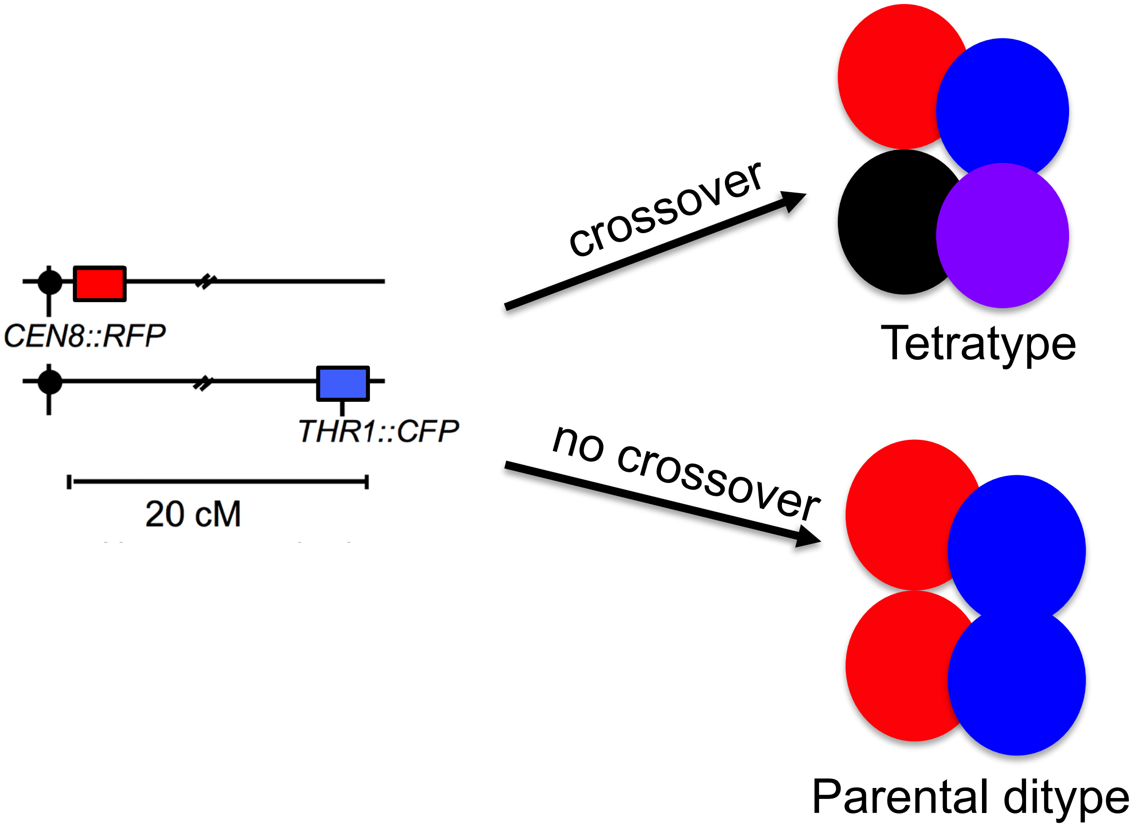

Supplementary Fig. 2. Spore autonomous fluorescence assay to monitor crossover frequencies. Diploid yeast bearing mutant *mlh3* alleles and indicated red (RFP) and cyan (CFP; colored blue in cartoon) fluorescence markers knocked into loci on Chr VIII are sporulated. Sister chromatids not shown. Segregation of single fluorescence-positive spores (blue or red) indicates no crossovers. Double positive (purple; lower right circle in the tetratype example) or fluorescence-negative spores (black circle) is indicative of a crossover.

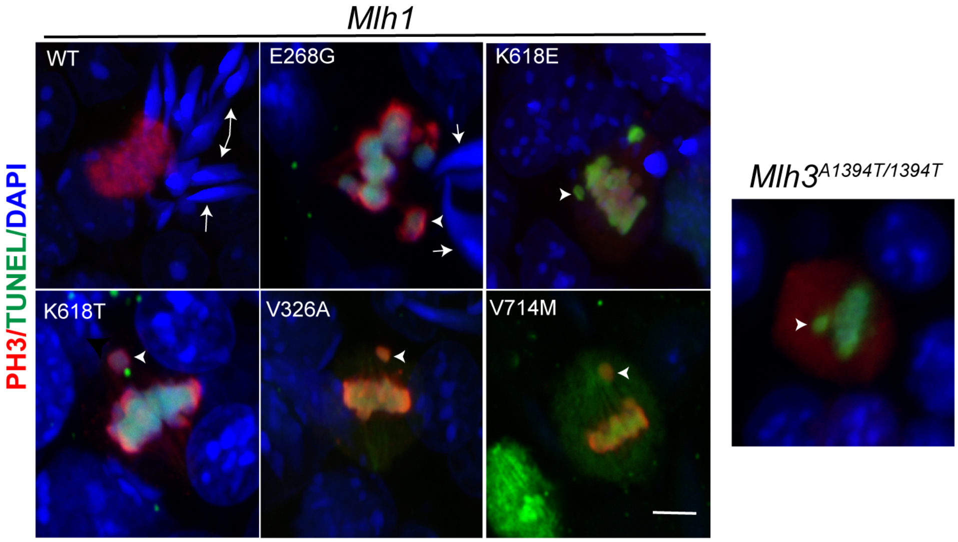

Supplementary Fig 3. Confocal projected images of MI spermatocytes illustrating misaligned chromosomes. Testes cross sections stained with antibody against phosphorylated histone H3 (Ser10) (red) and TUNEL (green) to visualize apoptotic metaphase cells. Arrowheads point to misaligned chromosomes. Arrows indicate elongated spermatids. Scale bar, 5μM.

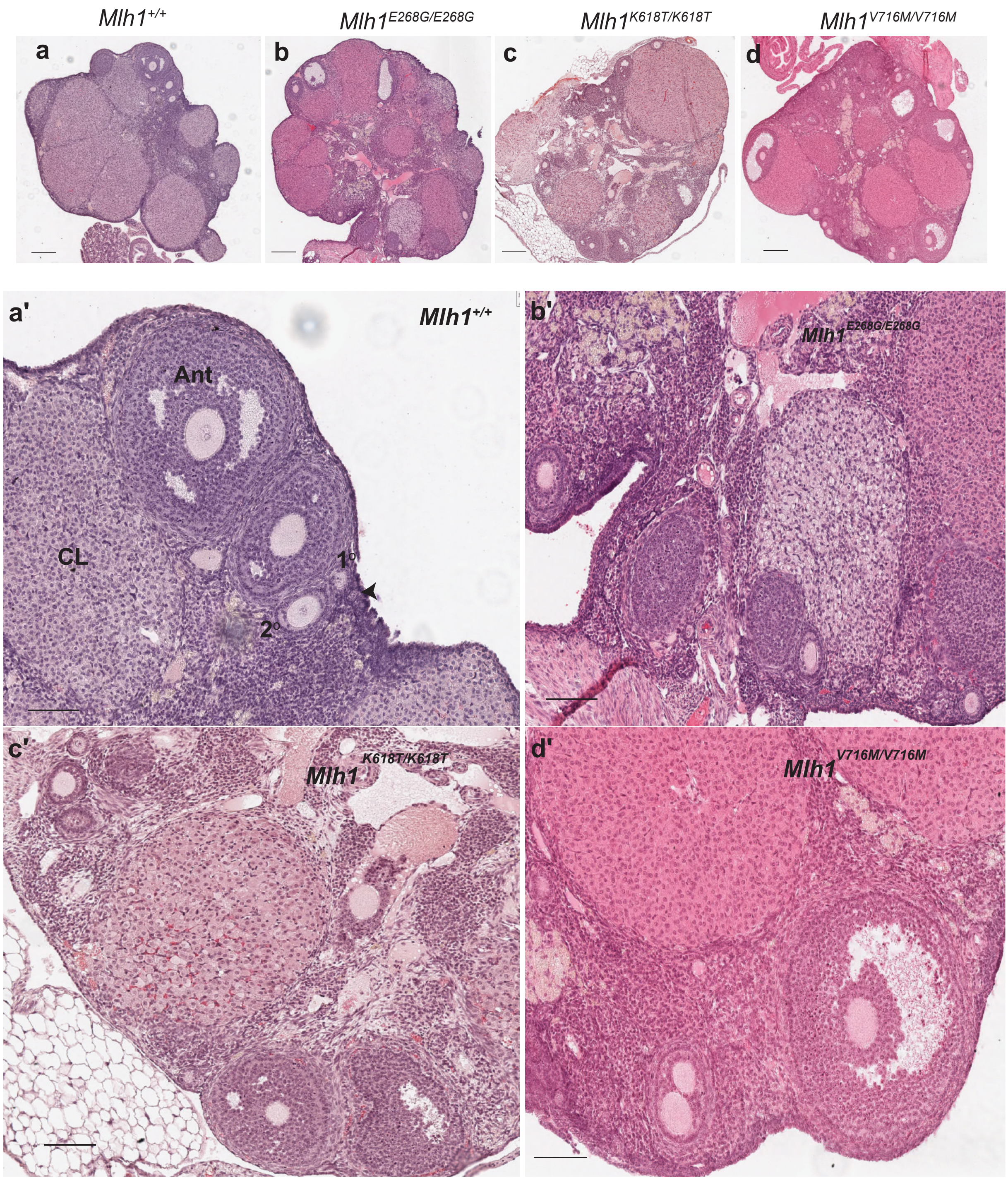

Supplementary Fig. 4. *MLH1* SNPs do not abolish ovulation in aged WT and *Mlh1* mutant females. H&E stained mouse ovaries from >11 month old females of indicated genotypes (abbreviation consistent with previous figures). Scale bar= 200μm in a-d and 100 μm in a'-d'. An- Antral follicle; 1º- primary follicle; 2º-secondary follicle; CL- Corpus Luteum; arrowhead- primordial follicle.

Supplementary Table 1a Analysis of yeast equivalents to human *mlh3* SNPs

	---*lys2-A₁₄* reversion rate---						Phenotype	
***mlh3* allele**	**(x 10^{-6})**	**95% CI (x 10^{-6})**	**n**	**relative to WT**	**% tetratype (n)**	**relative to WT**	**MMR**	**CO**
MLH3	3.70	1.27-6.80	14	1	39.9 (1052)	1	+	+
MLH3::KANMX#					39.8 (1004)	1.0		+
mlh3Δ	23.1	15.1-29.4	16	6.24	18.4 (706)	0.49	-	-
T65R	4.60	3.51-10.8	11	1.24	36.4 (272)*	0.97	+	+
R96Q	11.9	8.75-16.7	16	3.21	22.8 (302)**	0.61	+/-	-
R96G	not tested				21.8 (1122)**	0.58		-
P199L	12.9	9.77-17.8	16	3.49	23.1 (312)**	0.62	+/-	-
L313K	6.34	4.24-9.29	11	1.71	31.2 (266)*	0.83	+	+
R407I	12.7	5.20-17.3	16	3.43	38.9 (841)*	1.04	+/-	+
R530C	18.1	11.2-29.1	11	4.89	21.3 (258)**	0.57	-	-
R530H	17.7	13.1-19.2	11	4.78	19.9 (246)**	0.53	-	-
R532C	10.1	7.37-13.8	16	2.73	36.5 (537)*	0.97	+/-	+
A702T	9.06	6.63-10.9	16	2.45	24.6 (666)**	0.66	+/-	-
G704W	8.64	6.82-14.6	11	2.34	30.4 (642)***	0.81	+/-	+/-

Legend - *lys2-A₁₄* reversion (n= number of independent assays) and meiotic crossover analyses. For % tetratype (two spores containing parental, and two containing nonparental information, indicative of a crossover event), n represents the sum of parental ditype and tetratype events. For phenotypes: +, wild-type; -, null; +/-, intermediate. *p<0.0001 compared to null, p>0.002, compared to wild-type; **p>0.002 compared to null, p<0.0001 compared to WT; ***p<0.0001 compared to null, p<0.0001 compared to WT. A Bonferroni correction was applied for the Pearson Chi-Squared contingency test of the meiotic crossover data. 26 comparisons were made (13 to *MLH3::KANMX*, 13 to *mlh3Δ*), for a p<0.002 (0.05/26) cut off for statistical significance. # No difference in the reversion rate at the *lys2A₁₄* locus was observed between *MLH3* and *MLH3::KANMX* strains (Nishant et al., *Genetics* 179: 747, 2008).

Supplementary Table 1b Statistics for meiotic crossover assay in Supplementary Table 1a

***mlh3* allele**	**PD**	**TT**	**PD+TT**	**%TT**	**Pvalue to *MLH3::KANMX***	**Pvalue to null**	**Phenotype**
MLH3	635	417	1052	39.9	0.92	<0.0001	+
MLH3::KANMX	604	400	1004	39.8	**1**	<0.0001	+
mlh3null	576	130	706	18.4	<0.0001	**1**	-
T65R	173	99	272	36.4	0.30	<0.0001	+
R96G	877	245	1122	21.8	<0.0001	0.078	-
R96Q	233	69	302	22.8	<0.0001	0.11	-
P199L	240	72	312	23.1	<0.0001	0.085	-
L313K	183	83	266	31.2	0.0099	<0.0001	+
R407I	514	327	841	38.9	0.67	<0.0001	+
R530C	203	55	258	21.3	<0.0001	0.31	-
R530H	197	49	246	19.9	<0.0001	0.60	-
R532C	341	196	537	36.5	0.20	<0.0001	+
A702T	502	164	666	24.6	<0.0001	0.005	-
G704W	447	195	642	30.4	<0.0001	<0.0001	+/-

P-value: Pearson Chi-Squared contingency test, Vassar Stats, with a Bonferroni correction for 26 comparisons, for a p<0.002 cut off for statistical significance.

Supplementary Table 2. Yeast strains used in this study.

Strain	Genotype	Plasmid
EAY3252	*MATα, ho::hisG, ura3, leu2::hisG, trp1::hisG, ADE2, HIS4, CEN8Tomato::LEU2, MLH3, lys2::insE-*A_{14}	
EAY4480	*MATα, ho::hisG, ura3, leu2::hisG, trp1::hisG, ADE2, HIS4, CEN8Tomato::LEU2, MLH3::KANMX, lys2::insE-*A_{14}	
EAY3255	*MATα, ho::hisG, ura3, leu2::hisG, trp1::hisG, ADE2, his4xB, CEN8Tomato::LEU2, mlh3Δ::NATMX, lys2::insE-*A_{14}	
EAY3486	*MATa, ho::LYS2; lys2; ura3; leu2::hisG; trp1::hisG; THR1::m-Cerulean-TRP1; mlh3Δ::NATMX*	
EAY3944-3945	Same as EAY3255, but *mlh3-R530C::KANMX*	pEAI409
EAY3946-3947	Same as EAY3255, but *mlh3-R530H::KANMX*	pEAI410
EAY3948-3949	Same as EAY3255, but *mlh3-L313K::KANMX*	pEAI411
EAY3950-3951	Same as EAY3255, but *mlh3-T65R::KANMX*	pEAI412
EAY3953, 3982, 3983	Same as EAY3255, but *mlh3-R96Q::KANMX*	pEAI413
EAY4133, 4134	Same as EAY3255, but *mlh3-R96G::KANMX*	pEAI441
EAY3954-3955	Same as EAY3255, but *mlh3-R532C::KANMX*	pEAI414
EAY3956-3957	Same as EAY3255, but *mlh3-G704W::KANMX*	pEAI415
EAY3958-3959	Same as EAY3255, but *mlh3-R407I::KANMX*	pEAI416
EAY3960-3961	Same as EAY3255, but *mlh3-A702T::KANMX*	pEAI417
EAY3979-3981	Same as EAY3255, but *mlh3-P199L::KANMX*	pEAI418

All strains are from the SK1 background. "Plasmid" refers to the *mlh3::KANMX* vector used to integrate the indicated *mlh3* allele.

Supplementary Table 3. CRISPR and genotyping oligonucleotides

Allele	Forward primer	HR ssODN	Genotyping primers (5'-3') primer	primer
MLH3 R93G	**GAAATTAATACGACTCACTATAGG**GCAAGTATAGCCGACATGGC**GTTTTAGAGCTAGAAATAGC**	AGCATTTTATGGCTTCGGAGGAGAGGCCTTGGCAAGTATAGCCGACATGGCTGGTGCTGTGGAGATTTCATCCAAGAAAAACACAACACTGAAAA	ATGTGTGGCCATCAGAGTGA	CTGCCCGAACTTTCTCAAAC
MLH3 R1230H	**GAAATTAATACGACTCACTATAGG**GCTCCAAACGAATGCGTTCA**GTTTTAGAGCTAGAAATAGC**	CTTAAATCTCCTCTAACTACAGACAGATACTTACCAGTAATAAGCTGCTCCAAACGTATGTGTTCATGTGCAGCATGCTGGTCCACCAGGACTAACAGGTTTC CACCTACAAAATAATCC	GAGCACACAGCAAGGAGGTT	TCCTGGATTATTTTGTAGGTGGA
MLH3 A1394T	**GAAATTAATACGACTCACTATAGG**GTCAGCTAAGGGCAGCATTG**GTTTTAGAGCTAGAAATAGC**	GAAGAGAGCTGCCGCCTCATCGAAGCTCTGTCCTTGTCCCAGCTGCCATTTCAGTGTACTCATGGGAGACCATCTATGCTGCCCTTAGCTGACCTGGACCACTTGGAGCAGGAAAAACAG	CCAAGTGTGTTTTGGTTGGT	TGCACTGTAACTTTTTGGTGTC
MLH1 E268G	**GAAATTAATACGACTCACTATAGG**AGGCAGCTGATTCTACCAGA**GTTTTAGAGCTAGAAATAGC**	AATGGGTGTGTGTTTTTGGGCAAGTATGCTGCATATACAGTTTCAATGGCTTTTCTCAAGGCAGCTGATCCTACGAGTCGGTCTGAAATCTGAAAAATCAAAAGGAGAGTTAACCAGATCACACTTG	CCTGGCTGACAACCTCTTTC	GCTGAAGGTCAATGACAGCA
MLH1 K618E	**GAAATTAATACGACTCACTATAGG**AGGACGACGGCCCGAAGGAA**GTTTTAGAGCTAGAAATAGC**	AAGTGGCTGGACAGAGGACGACGGCCCGAAAGAGGGCCTTGCAGAGTACATTGTTGAGTTTCTGAAGAAGGAAGCGGAGATGCTTGCAGACTATTTCTCTGTGGAGATCGATGAGGCGAG	TGTCCCAACCTAGGGACTTG	AGGGCTATTGAAACCAGCAC
MLH1 K618T	**GAAATTAATACGACTCACTATAGG**AGGACGACGGCCCGAAGGAA**GTTTTAGAGCTAGAAATAGC**	AAGTGGCTGGACAGAGGACGACGGCCCGAAAGAGGGCCTTGCAGAGTACATTGTTGAGTTTCTGAAGAAGACAGCGGAGATGCTTGCAGACTATTTCTCTGTGGAGATCGATGAGGCGAG	TGTCCCAACCTAGGGACTTG	AGGGCTATTGAAACCAGCAC
MLH1 V326A	**GAAATTAATACGACTCACTATAGG**GCACATTGAGAGCAAGCTGC**GTTTTAGAGCTAGAAATAGC**	AAGCACGAAGTTCACTTTCTGCACGAGGAGAGCATTCTGCAGCGTGCGCAGCAGCACATTGAGAGCAAGCTCCTCGGCTCCAATTCCTCCAGGATGTATTTCACCCAG	CATGTGCATGGAACACACAG	TCTAGTCGCCACATGTCACC
MLH1 V716M	**GAAATTAATACGACTCACTATAGG**CCACTTCCAGGGCTTTGACG**GTTTTAGAGCTAGAAATAGC**	TGTGAAATGCTTCGGAGGTAGGAGGTGTGAGCGGAAGGCTTTATAGATAATGTGCTCCATAGTCCACTTCCAGGGCTTAGAGGTCGAGCCAGGCATGTCACTCTGGAAAATATATGACAC	AAAATCACACCAATACACGTGAG	TGCAGAGGCTGCTTTGTAAG
MLH1 K618A	**GAAATTAATACGACTCACTATAGG**AGGACGACGGCCCGAAGGAA**GTTTTAGAGCTAGAAATAGC**	AAGTGGCTGGACAGAGGACGACGGCCCGAAAGAGGGCCTTGCAGAGTACATTGTTGAGTTTCTGAAGAAGGCAGCGGAGATGCTTGCAGACTATTTCTCTGTGGAGATCGATGAGGCGAG	TGTCCCAACCTAGGGACTTG	AGGGCTATTGAAACCAGCAC

Note: The reverse primer for making sgRNA by overlap PCR was the same in all cases: gcaccgactcggtgccactttttcaagttgataacggactagccttattttaacttgctatttctagctctaaaac
