# Supplementary material for: Human MLH1/3 variants causing aneuploidy, pregnancy loss, and premature reproductive aging
Source: Nat Commun. 2021 Aug 18;12:5005. doi: 10.1038/s41467-021-25028-1 (PMC8373927; doi:10.1038/s41467-021-25028-1)
Supplement: Supplementary file 4 — Description of additional supplementary files [file 41467_2021_25028_MOESM4_ESM.docx]

Description of additional supplementary files

Title: Supplementary Table 1

Description: PPH2 = Polyphen 2; nucleotide positions are from hg19. The protein model corresponds to UniPot P40692. The alleles shaded gray were selected for mouse modeling.
