# Supplementary material for: Human MLH1/3 variants causing aneuploidy, pregnancy loss, and premature reproductive aging
Source: Nat Commun. 2021 Aug 18;12:5005. doi: 10.1038/s41467-021-25028-1 (PMC8373927; doi:10.1038/s41467-021-25028-1)
Supplement: Supplementary file 5 — Reporting summary1 [file 41467_2021_25028_MOESM5_ESM.pdf]

Corresponding author(s): John Schimenti

Last updated by author(s): June 29, 2021

Reporting Summary

Nature Research wishes to improve the reproducibility of the work that we publish. This form provides structure for consistency and transparency in reporting. For further information on Nature Research policies, see our Editorial Policies and the Editorial Policy Checklist.

Statistics

For all statistical analyses, confirm that the following items are present in the figure legend, table legend, main text, or Methods section.

n/a	Confirmed	
☐	☒	The exact sample size (*n*) for each experimental group/condition, given as a discrete number and unit of measurement
☐	☒	A statement on whether measurements were taken from distinct samples or whether the same sample was measured repeatedly
☐	☒	The statistical test(s) used AND whether they are one- or two-sided<br>*Only common tests should be described solely by name; describe more complex techniques in the Methods section.*
☒	☐	A description of all covariates tested
☒	☐	A description of any assumptions or corrections, such as tests of normality and adjustment for multiple comparisons
☐	☒	A full description of the statistical parameters including central tendency (e.g. means) or other basic estimates (e.g. regression coefficient) AND variation (e.g. standard deviation) or associated estimates of uncertainty (e.g. confidence intervals)
☐	☒	For null hypothesis testing, the test statistic (e.g. *F*, *t*, *r*) with confidence intervals, effect sizes, degrees of freedom and *P* value noted<br>*Give P values as exact values whenever suitable.*
☒	☐	For Bayesian analysis, information on the choice of priors and Markov chain Monte Carlo settings
☒	☐	For hierarchical and complex designs, identification of the appropriate level for tests and full reporting of outcomes
☒	☐	Estimates of effect sizes (e.g. Cohen's *d*, Pearson's *r*), indicating how they were calculated

Our web collection on statistics for biologists contains articles on many of the points above.

Software and code

Policy information about availability of computer code

Data collection	Clones for MLH1 and all Y2H-tested interaction partners were obtained from hORFeome v8.1. To select MLH1 SNPs for cloning and subsequent Y2H testing, we used gnomAD v2.1.
Data analysis	We did not use any custom code.<br>Graphs and statistical analyses were performed with GraphPad Prism5.<br>Following identical background adjustments for all images, cropping, color, and contrast adjustments were made with Adobe Photoshop CC 2017.<br>Eggs were imaged with the i880 (Zeiss) confocal at 0.5 μm z-intervals. Chromosome counting was performed with NIH Image J software, using cell counter plugins.

For manuscripts utilizing custom algorithms or software that are central to the research but not yet described in published literature, software must be made available to editors and reviewers. We strongly encourage code deposition in a community repository (e.g. GitHub). See the Nature Research guidelines for submitting code & software for further information.

Data

Policy information about availability of data

All manuscripts must include a data availability statement. This statement should provide the following information, where applicable:

- Accession codes, unique identifiers, or web links for publicly available datasets
- A list of figures that have associated raw data
- A description of any restrictions on data availability

All the relevant quantitative data is present in the manuscript. Image files (for example, immunostaining) underlying quantifications are retained by the laboratories.

Field-specific reporting

Please select the one below that is the best fit for your research. If you are not sure, read the appropriate sections before making your selection.

☒ Life sciences ☐ Behavioural & social sciences ☐ Ecological, evolutionary & environmental sciences

For a reference copy of the document with all sections, see nature.com/documents/nr-reporting-summary-flat.pdf

Life sciences study design

All studies must disclose on these points even when the disclosure is negative.

Sample size	>3 animals per data point were analyzed which is standard in the field.
Data exclusions	No data was excluded.
Replication	Data is from at least 3 biological replicates that were successful.
Randomization	Generally, there were two groups: mutant vs non-mutant....these are not random.
Blinding	Blinding was not performed... for yeast or mice experiments.

Reporting for specific materials, systems and methods

We require information from authors about some types of materials, experimental systems and methods used in many studies. Here, indicate whether each material, system or method listed is relevant to your study. If you are not sure if a list item applies to your research, read the appropriate section before selecting a response.

Materials & experimental systems

n/a	Involved in the study
☐	☒ Antibodies
☒	☐ Eukaryotic cell lines
☒	☐ Palaeontology and archaeology
☐	☒ Animals and other organisms
☒	☐ Human research participants
☒	☐ Clinical data
☒	☐ Dual use research of concern

Methods

n/a	Involved in the study
☒	☐ ChIP-seq
☒	☐ Flow cytometry
☒	☐ MRI-based neuroimaging

Antibodies

Antibodies used	: rabbit anti-SYCP3 (1:500, ab15093; Abcam), mouse anti-SYCP3 (1:500, ab97672; Abcam), and mouse anti-MLH1 (1:50, 550838; BD Pharmingen), the anticentromere antibody (ACA) to detect centromeres (Antibodies Incorporated; #15-234; 1:30) . Secondary antibodies were goat anti rabbit-IgG 488 (1:2,000, A11008; Molecular Probes), goat ant-rabbit IgG 594 (1:1,000, A11012; Molecular Probes), and goat anti-mouse IgG 594 (1:1,000, A11005; Molecular Probes). anti-pH3 (Ser10), 1:100, Millipore). Unfortunately, because the analyses were performed over many years, lot numbers were not recorded.
Validation	Manufacturer website links that shows multiple cross references:<br>SYCP3 (ab15093)- https://www.abcam.com/scp3-antibody-ab15093.html<br>SYCP3 (ab97672)- https://www.abcam.com/scp3-antibody-cor-10g117-ab97672.html<br>MLH1 (550838; BD Pharmingen)- https://www.bdbiosciences.com/ds/pm/tds/550838.pdf<br>anticentromere antibody (ACA) (Antibodies Incorporated; #15-234; 1:30)<br><br>These antibodies have well-defined patterns of staining during meiosis.

Animals and other organisms

Policy information about studies involving animals; ARRIVE guidelines recommended for reporting animal research

Laboratory animals	Mus musculus, males and females, 3 weeks to 12 month old animals were used Strains used: C57BL/6J and FvB/nJ
Wild animals	This study did not involve wild animals
Field-collected samples	This study did not involve the samples collected from field

Ethics oversight

Experiments with the animals were performed under a protocol (2004-0038) approved by Cornell’s Animal Care and Use Committee

Note that full information on the approval of the study protocol must also be provided in the manuscript.
